# Mutation testing in melanoma families: INK4A, CDK4 and INK4D

**DOI:** 10.1038/sj.bjc.6690354

**Published:** 1999-04

**Authors:** J A Newton Bishop, M Harland, D C Bennett, V Bataille, A M Goldstein, M A Tucker, B A J Ponder, J Cuzick, P Selby, D T Bishop

**Affiliations:** ICRF Genetic Epidemiology, Leeds, UK; ICRF Cancer Medicine Research Unit, St James's University Hospital, Beckett Street, Leeds LS9 7TF, UK; St George's Hospital Medical School, London, UK; ICRF Skin Tumour Laboratory, Royal London Hospital, London, UK; Genetic Epidemiology Branch, National Cancer Institute, Bethesda, MD, USA; CRC Human Cancer Genetics Group, Box 238, Addenbrooke's Hospital, Cambridge, UK; ICRF Mathematics Statistics and Epidemiology, Lincolns Inn Fields, London, UK

**Keywords:** INK4A, INK4D, familial melanoma

## Abstract

The *INK4A* gene which codes for the cyclin-dependent kinase (CDK) inhibitor INK4A or p16 underlies susceptibility to melanoma in some families. Germline mutations in the gene that codes for the target protein of p16, CDK4, underlie susceptibility in very rare families. We report mutation screening of the *INK4A* and *CDK4* genes in 42 UK families. A total of nine families were identified with *INK4A* mutations and none with *CDK4* exon 2 mutations. These mutations were in 8/22 (35%) families with three or more cases of melanoma and 1/20 (5%) families with only two cases. In one of these nine families a novel single base pair substitution was identified, Gly67Arg. In an attempt to identify another melanoma susceptibility gene, a member of the *INK4* family, the *p19 INK4D* gene has been studied. The *p19* gene was sequenced in DNA from the 42 UK families and six additional US families. No mutations were identified. © 1999 Cancer Research Campaign


*British Journal of Cancer* (1999) **80**(1/2), 295–300
© 1999 Cancer Research Campaign
Article no. bjoc.1998.0354

### Mutation testing in melanoma families: INK4A, CDK4 and INK4D

**JA Newton Bishop[2], M Harland[1], DC Bennett[3], V Bataille[4], AM Goldstein[5], MA Tucker[5], BAJ Ponder[6], J Cuzick[7], P Selby[2] and DT Bishop[1]**

[1]ICRF Genetic Epidemiology, Leeds, UK; [2]ICRF Cancer Medicine Research Unit, St James's University Hospital, Beckett Street, Leeds LS9 7TF, UK; [3]St George's Hospital Medical School, London, UK; [4]ICRF Skin Tumour Laboratory, Royal London Hospital, London, UK; [5]Genetic Epidemiology Branch, National Cancer Institute, Bethesda, MD, USA; [6]CRC Human Cancer Genetics Group, Box 238, Addenbrooke's Hospital, Cambridge, UK; [7]ICRF Mathematics Statistics and Epidemiology, Lincolns Inn Fields, London, UK

**Summary** The *INK4A* gene which codes for the cyclin-dependent kinase (CDK) inhibitor INK4A or p16 underlies susceptibility to melanoma in some families. Germline mutations in the gene that codes for the target protein of p16, CDK4, underlie susceptibility in very rare families. We report mutation screening of the *INK4A* and *CDK4* genes in 42 UK families. A total of nine families were identified with *INK4A* mutations and none with *CDK4* exon 2 mutations. These mutations were in 8/22 (35%) families with three or more cases of melanoma and 1/20 (5%) families with only two cases. In one of these nine families a novel single base pair substitution was identified, Gly67Arg. In an attempt to identify another melanoma susceptibility gene, a member of the *INK4* family, the *p19 INK4D* gene has been studied. The *p19* gene was sequenced in DNA from the 42 UK families and six additional US families. No mutations were identified.

**Keywords:** INK4A; INK4D; familial melanoma

Rare families exist in which there appears to be a predisposition to cutaneous melanoma (Clark et al, 1978; Newton, 1994). In some families, there is an apparent susceptibility to other tumours, particularly to pancreatic (Lynch and Fusaro, 1991) and other gastrointestinal cancers (Bergman et al, 1990), but in most families the susceptibility appears to be to melanoma alone. Early linkage data suggestive of linkage to chromosome 1 (Bale et al, 1989; Goldstein et al, 1993) have not been substantiated (Gruis et al, 1990; Nancarrow et al, 1992), but strong evidence of linkage to chromosome 9 (Cannon-Albright et al, 1992; Nancarrow et al, 1993) was quickly followed by the identification of germline mutations in the *INK4A* (*CDKN2*) gene (on chromosome 9p) which codes for the p16 protein, in melanoma families (Hussussian et al, 1994; Kamb et al, 1994). Mutations in this gene have been identified in the germline of families world-wide (Dracopoli and Fountain, 1996), mutations that co-segregate with cases and for which abnormal CDK4 binding has been demonstrated (Harland et al, 1997). The proportion of families with mutations, however, remains moderate, at around 40% of families with three or more cases overall. In our initial UK series, five of 12 families of this type, and one of 12 families with two cases of melanoma had identifiable mutations in the *INK4A* gene (Harland et al, 1997).

There is some evidence for linkage to chromosome 9 even in families without identifiable *INK4A* mutations (Hussussian et al, 1994; Liu et al, 1997) so that there may be additional mutations yet to be identified in the promoter region of the gene, or in other linked genes. No significant contribution has been identified from two candidate genes in this region, coding for the protein p15 and p14ARF (Fitzgerald et al, 1996). There are, however, haplotype data to suggest that there are likely to be other melanoma susceptibility genes elsewhere (Goldstein et al, 1994; MacGeoch et al, 1994).

Zuo et al (1996) have identified one such gene. They reported mutations in the gene that codes for the target protein of p16, *CDK4*. Three families have now been reported world-wide in which germline *CDK4* mutations have been detected in melanoma families (Zuo et al, 1996; Bressac-de-Paillerets et al, 1997). In these families, single base pair substitutions were detected at the same point in the p16-binding domain, residue 24 and, more recently, mutations have been detected in a second domain also important in binding to cyclin D1 (Coleman et al, 1997), strengthening considerably the significance of the p16–CDK4–RB pathway in melanoma carcinogenesis. Several groups have now looked at their melanoma families without identifiable *INK4A* mutations and have failed to identify additional *CDK4* mutations. It is clear, therefore, that *CDK4* mutations are rare (Harland et al, 1997) and that there are other melanoma susceptibility genes still to identify.

p16 is a member of a family of CDK inhibitors, the INK4 family. The two cyclin D-dependent CDKs, CDK4 and CDK6, induce phosphorylation of the retinoblastoma protein (RB1), thereby releasing RB1 from its growth inhibitory functions. In turn, the proteins from the INK4 family inhibit this process and act as brakes to phase G1 of the cell cycle (Hirama and Koeffler, 1995; Sherr and Roberts, 1995; Sherr, 1996). There are at least four members of the family, p16INK4A, p15INK4B, p18INK4C and p19INK4D (to be distinguished from p14ARF). The

*Received 24 August 1998*
*Revised 16 October 1998*
*Accepted 4 November 1998*

*Correspondence to:* JA Newton Bishop

The first two authors contributed equally to this work.

**Table 1** *p19 INK4D* primers used

| Primer | Sequence | Annealing temp (°C) | Usage |
|---|---|---|---|
| p19-N1F | GCCAGTGTCGACATGCTG | 55 | Long-range PCR (F)<br>Intronic sequencing (F)<br>PCR amplification, exon 1 (F) |
| p19-N2R | TGTCCAACACACCAAAAGGA | 55 | Long-range PCR (R) |
| p19-2R | CGGAGGTGTCCTGGACATTG | 55 | Intronic sequencing (R) |
| p19-in1F | CTGGGAACCGGTCCTTCTCT | 55 | Intronic sequencing (F) |
| p19-in1R | AGGGGTCACTGGAAAGAGAA | 55 | Intronic sequencing (R) |
| p19-ex1R | CTGGGAACCGGTCCTTCTCT | 55 | PCR amplification, exon 1 (R) |
| p19-143F | ACTTTCCTGGGGGCTTATGC | 55 | PCR amplification, exon 2 (F) |
| p19+98R | CTATAAGCCACAAACTGTGC | 55 | PCR amplification, exon 2 (R) |

expression of the *INK4* genes shows tissue specificity suggesting lack of redundancy (Sherr, 1996). The polypeptides are composed of 4–5 ankyrin repeats, and mutagenesis studies have suggested that mutations are likely to affect function only if they occur in the region coding for these ankyrin repeats (Yang et al, 1996). There is a little information which suggests that the INK4 family members may have a role in cellular differentiation via effects of the CDKs on phosphorylation of substrates other than RB; for example, p18 and p19 which are widely expressed during embryogenesis (Zindy et al, 1997).

All members of the family bind to CDK4 and 6 (Hirai et al, 1995; Guan et al, 1996). They have a moderate degree of homology. p19INK4D, for example, has 48% amino acid homology with p16INK4A. All members of the family compete with cyclin D1 for its binding site on CDKs (Guan et al, 1996). The presence of each member of the family in phase G1 will induce G1 arrest, so that at a cellular level at least all members of the family are potential tumour suppresser genes. We chose *p19 INK4D* as a candidate gene because of its chromosomal location at 19p13.2, a reported site of repeated translocation in melanoma tumours (Parmiter et al, 1986).

### MATERIALS AND METHODS

DNA from 42 UK families at increased risk of melanoma was screened for germline mutations. The families have been recruited in the period since 1989 in England and Wales. Ethical Committee approval for this study was obtained from all institutions involved. Twenty-two of these families have been previously described (Harland et al, 1997). Wherever possible, DNA from two cases from each family was screened. Six additional US families were screened for *INK4D* mutations. These families were selected because they had sarcoma and/or haematological malignancies in addition to melanoma. Three of these families have been previously described (Goldstein et al, 1994; Liu et al, 1997).

### DNA extraction and PCR

DNA extraction from peripheral blood, and the polymerase chain reaction (PCR) amplification of the three coding exons of *INK4A*, and exon 2 of *CDK4*, was carried out as described previously (Harland et al, 1997).

### Analysis of *p19 INK4D*

Previously, only the cDNA sequence has been available for *p19 INK4D* (GenBank accession numbers U40343, U20498 and U49399). Studies using primers designed from this sequence have been able to cover, at best, 85% of the coding sequence of *INK4D* (Shiohara et al, 1996; Miller et al, 1997). In order to examine the terminal amino acids in each of the two exons of *INK4D*, and also the splice donor and acceptor sites, it was important to determine the intronic sequence of *INK4D*, so that intronic primers flanking the two coding exons could be designed. Primers flanking the cDNA sequence were designed from the available sequence for the 3′ and 5′ non-coding regions (Guan et al, 1996) (GenBank accession number U40343). Long-range PCR across the two exons and the intron, in genomic DNA samples, produced a PCR product approximately 1100 bp longer than would be predicted from the cDNA sequence. Sequencing through the coding regions and into the intron has enabled us to determine the complete intronic sequence of *INK4D*, 1091 bases in total. This sequence, which has been submitted to GenBank (accession number AF061327), has enabled us to design primers flanking the two exons of p19, and so allowed us to investigate the complete coding region of *p19 INK4D* in our families.

### Long-range PCR of *p19 INK4D*

Primers were based on the *p19 INK4D* cDNA sequence (GenBank accession number U40343) (Table 1). A total of 100 μl long-range PCR reactions were carried out using 250 ng genomic DNA, 0.2 mM dNTPs, 50 μM each primer, 10% dimethyl sulphoxide (DMSO), and 5 units Deep Vent DNA polymerase (New England Biolabs), in the reaction buffer provided by the supplier. Thermal cycling conditions were as follows: an initial denaturation at 94°C for 5 min; followed by 35 cycles of denaturing at 94°C (30), annealing at 55°C (30), and extension at 72°C (6′), with a final 10-min extension at 72°C. PCR fragments were isolated by agarose gel electrophoresis and purified prior to sequencing using the QIAquick Gel Extraction Kit (Quiagen). Sequencing of the intronic region was performed as described below, using forward and reverse primers designed from the known cDNA sequence, and also primers designed from generated intronic sequence (Table 1). PCR amplification for the screening of the two exons of *p19 INK4D*, was carried out as described previously for *INK4A* and *CDK4* (Harland et al, 1997), the primers used are shown in Table 1.

### Sequencing

Sequencing reactions were carried out using the ABI PRISM BigDye Terminator Cycle Sequencing Kit (Perkin-Elmer) and the

 © Cancer Research Campaign 1999

products were analysed on an ABI 377 DNA sequencer. DNA sequencing was performed in both directions, initiated from the forward and reverse primers used in the initial PCR amplification of each exon. In kindreds where sequence variation was found in one or both of the initial samples selected for investigation, all members of that family, for whom DNA was available, were sequenced for the putative mutation identified in the initial samples, in order to study the segregation of the mutation with melanoma.

### RESULTS

Table 2 shows the characteristics of the families screened. Forty-two UK families were mutation tested (Table 3). Partial testing was reported in 22 of these families previously (Harland et al, 1997). Six additional US families were screened for *INK4D* mutations in whom significant *INK4A* and *CDK4* mutations had previously been excluded (Hussussian et al, 1994). In one of these families, Asn71Ser was identified. This mutation has not yet been shown to have functional implications (Ranada et al, 1995). In total, 26 families with three or more cases, and 22 with only two cases of melanoma were screened. In nine of the 22 families with two cases of melanoma, one of those cases had proven multiple primary tumours, as additional evidence for an underlying genetic susceptibility to melanoma.

The three UK families not previously reported with germline mutations had Arg24Pro (in common with another UK family and several others world-wide), Met53Ile (again in common with a second UK family and others world-wide) and a novel single base pair substitution Gly67Arg (Figure 1).

A previously reported polymorphism in exon 1 of *INK4D*, was detected in codon 30 (Miller et al, 1997) in cases from five families with three or more cases of melanoma and one family with two cases only. No mutations were identified in either *CDK4* exon 2 or the *INK4D* genes.

### DISCUSSION

*INK4A* (*CDKN2*) clearly underlies susceptibility to melanoma in a significant proportion of melanoma-prone families, and germline mutations in the *CDK4* gene underlie susceptibility in a small minority of families. We report three new families with germline mutations. Two of these have been widely reported in other centres, and in all families the mutations co-segregate with the tumours. Furthermore, functional assays have shown these mutations to be significant (Harland et al, 1997). The novel mutation Gly67Arg has not, to our knowledge, been reported before, but it occurs at a site predicted by the ankyrin structure of the p16 protein, to be critical to function. A similar, single base pair substitution at the same codon has been detected in an Australian pedigree (Mann et al, 1997).

In this series of 42 UK families recruited since 1989, 36% of families with three or more cases and 5% of families with only two cases had identifiable *INK4A* mutations. The prevalence of germline *INK4A* mutations in the larger families is consistent with that reported world-wide. Some authors have suggested that approximately 25% of families with two or more cases in Australia, Europe and the USA have identifiable mutations, and in a population-based study in Sweden by Platz et al the frequency of these mutations was estimated to be even lower (Platz et al, 1997).

**Table 2** The family characteristics of those families screened in which there were three or more cases of melanoma. The number of cases reported is shown, and whether or not the family is known to have had cases with multiple primaries

| Pedigree number | Number of cases | Cases with multiple primaries | *INK4A* exon 1 | *INK4A* exon 2 | *INK4A* exon 3 | *CDK4* exon 2 | *INK4D* exon 1 | *INK4D* exon 2 |
|---|---|---|---|---|---|---|---|---|
| MEL21 | 10 | Yes | Arg24Pro | | | | | |
| NIH 1 | 10 | Yes | | *Ala148Thr* | | | | |
| MEL07 | 5 | Yes | | | | | | |
| MEL09 | 5 | Yes | | Met53Ile | | | *90C/G* | |
| MEL32 | 5 | Yes | | Met53Ile | | | | |
| MEL48 | 5 | Not known | | | | | | |
| NIH 2 | 5 | Yes | | | | | *90C/G* | |
| MEL02 | 4 | Not known | | | | | *90C/G* | |
| MEL03 | 4 | No | | | | | | |
| MEL06 | 4 | Yes | | | | | | |
| MEL13 | 4 | Yes | 23ins24 | | | | | |
| MEL15 | 4 | Yes | | Ala118Thr | | | | |
| MEL26 | 4 | Yes | | | | | | |
| MEL37 | 4 | Not known | | | | | *90C/G* | |
| MEL39 | 4 | Not known | | Gly67Arg | | | | |
| MEL40 | 4 | Yes | Arg24Pro | | | | | |
| MEL49 | 4 | Yes | | | | | *90C/G* | |
| MEL24 | 3 | No | 88delG | | | | | |
| MEL28 | 3 | No | | | | | | |
| MEL30 | 3 | No | | | | | | |
| MEL35 | 3 | No | | | | | *90C/G* | |
| MEL44 | 3 | Yes | | | | | *90C/G* | |
| MEL50 | 3 | No | | | | | *90C/G* | |
| MEL53 | 3 | Yes | | | | | *90C/G* | |
| NIH 3 | 3 | Yes | | *Asn71Ser* | | | | |
| NIH 4 | 3 | No | | | | | | |

Polymorphisms are indicated in italics. The protein produced by mutation Asn71Ser has been normal in vitro testing.

*© Cancer Research Campaign 1999*

**Table 3** The family characteristics of those families screened in which there were two cases of melanoma. All families have been screened for exons 1–3 of *INK4A*, exon 2 of *CDK4* and exons 1 and 2 of *INK4D*. Only positive findings are indicated.

| Pedigree number | Number of cases | Cases with multiple primaries | *INK4A* exon 1 | *INK4A* exon 2 | *INK4A* exon 3 | *CDK4* exon 2 | *INK4D* exon 1 | *INK4D* exon 2 |
|---|---|---|---|---|---|---|---|---|
| MEL01 | 2 (1st) | Yes | | | | | | |
| MEL04 | 2 (2nd) | Yes | | | | | | |
| MEL11 | 2 (1st) | Yes | | | | | | |
| MEL14 | 2 (1st) | Yes | | | | | | |
| MEL16 | 2 (1st) | Yes | | | | | | |
| MEL19 | 2 (1st) | Yes | | | | | | |
| MEL20 | 2 (2nd) | No | | | | | | |
| MEL22 | 2 (1st) | Yes | | | | | | |
| MEL23 | 2 (1st) | Yes | | | | | | |
| MEL25 | 2 (2nd) | Not known | | | | | | |
| MEL29 | 2 (1st) | Not known | 23ins24 | | | | | |
| MEL34 | 2 (1st) | No | | | | | | |
| MEL38 | 2 (1st) | No | | | | | | |
| MEL41 | 2 (2nd) | No | | | | | | |
| MEL42 | 2 (1st) | No | | | | | | |
| MEL43 | 2 (1st) | No | | | | | | |
| MEL46 | 2 (1st) | No | | | | | | |
| MEL54 | 2 (1st) | Not known | | | | | | |
| MEL55 | 2 (1st) | No | | | | | | |
| MEL56 | 2 (1st) | No | | | | | | |
| NIH 5 | 2 (1st) | Yes | | | | | | |
| NIH 6 | 2 (1st) | No | | | | | | |

[a] Figure in brackets denotes the degree of relatedness between affected individuals.

**Table 4** A summary of the findings in 48 families. The decreasing likelihood of identifying a mutation with decreasing numbers of cases is illustrated

| Number of cases in family | Number of families | Number *INK4A* mutations found | Families with mutations (%) |
|---|---|---|---|
| 10 | 2 | 1 | 50 |
| 5 | 5 | 2 | 40 |
| 4 | 10 | 3 | 30 |
| 3 | 9 | 2 | 22 |
| 2 | 22 | 1 | 5 |

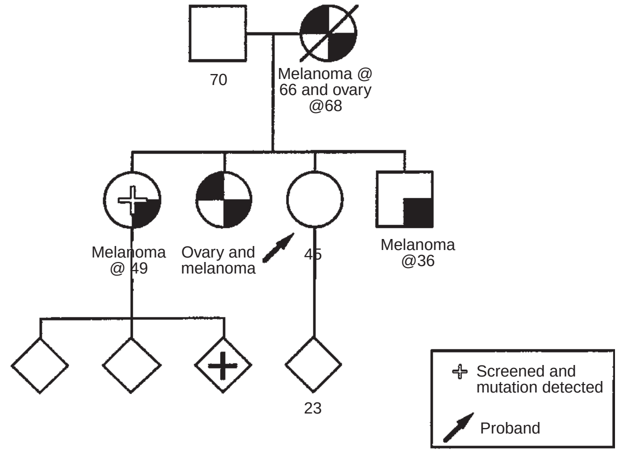

**Figure 1** Pedigree of family in which a germline mutation Gly67Arg was identified. In all, there were four cases of melanoma. The mutation was detected in two samples tested, one of which was taken from a melanoma case

The reported studies are difficult to compare, as noted by Goldstein and Tucker (1997), because different groups have been variably stringent in their selection criteria. Some stipulate that the number of cases reported should be in first-degree relatives, others do not. Furthermore, groups appear to vary in how rigorously they sought mutations. In this study, there appears to be a clear difference in the likelihood of identifying germline *INK4A* mutations in families of different sizes. Mutations were significantly more likely in families with larger numbers of cases (Table 4). This might imply that the families with larger numbers of cases are less likely to represent clustering of cases by chance alone, or that they carry more penetrant genes.

Overall, in the families with three or more cases, 64% did not have identifiable *INK4A* mutations, nor did they have the rarer *CDK4* exon 2 mutations. There is some evidence from other groups that some families without detectable *INK4A* mutations still have evidence of linkage to chromosome 9 (Walker et al, 1995) and it may be that, in these families, there are still to be found mutations in intronic sequences or the promoter that have an effect on p16 transcription.

There is some evidence for linkage to other chromosomes (Bale et al, 1989; Walker et al, 1994) and we have previously reported suggestive haplotype data (MacGeoch et al, 1994) so that it also seems likely that there are other high penetrance melanoma susceptibility genes to be identified. We have taken a candidate gene approach to the identification of such genes. It would seem that the p16/RB1 pathway is of critical importance in melanoma carcinogenesis and therefore we chose to look at another member of the INK4 family, *p19 INK4D*.

The four members of the INK4 family appear to function in a similar way in terms of binding to CDK4/6 and inducing G1 arrest. The role of members of the INK4 family, other than INK4A, in carcinogenesis is not yet clear. Mutations in all four members of

 *© Cancer Research Campaign 1999*

the family have been sought in cell lines. In one study of 100 cell lines for example, 35 homozygous deletions and seven intragenic mutations were detected in *INK4A*, and 29 lines had homozygous deletions in *INK4B*. Germline mutations have been sought both in the *INK4B* gene coding for p19 and in p14ARF (whose transcript is derived in part from an alternative codon reading frame of p16) but no evidence has yet been found for such mutations in melanoma families (Fitzgerald et al, 1996; Liu et al, 1997). INK4D is the most recently described member of the INK4 family and the gene is on chromosome 19 (19p13). Structural chromosome breakpoints are reported in around 4% of short-term melanoma cultures on 19p (Nelson et al, 1996). The chromosomal location of *INK4D* is, however, of most interest because of a report by Parmiter et al (1986) of a translocation involving chromosome 19 at 19p13 in three cases of melanoma. In only one line, an ovarian cancer cell line SKOV3, was there a single base pair deletion in exon 1 of *INK4D* (Gemma et al, 1996). In tumour samples, abnormalities have rarely been reported (Shiohara et al, 1996; Miller et al, 1997). These studies in cell lines and tumour samples suggest a possible role for *INK4D* in some specific tumour types but suggest that *INK4D* is not a tumour suppresser gene of such fundamental importance as *INK4A*.

In summary, we have not found any significant *INK4D* mutations in the germline of 48 families with a susceptibility to melanoma. Its similar function to that of INK4A and its chromosomal location at a site of translocations in melanoma patients, made it a candidate gene for genetic susceptibility to melanoma. To date we have no evidence for such a role, but it might reasonably be argued that if p19 does underlie susceptibility in some families, these families are likely to be rare and screening of samples from other continents should also be carried out.

### ACKNOWLEDGEMENTS

This work was funded by the Imperial Cancer Research Fund in the UK. Research nurses, Elizabeth Pinney and Patricia Mack, played a key role in collecting family data. BAJP is a Gibb Fellow of the Cancer Research Campaign. We are grateful to Dr PJ Morrison and Dr Trevor Cole who kindly referred us two families.

### REFERENCES

Bale SJ, Dracopoli NC, Tucker MA, Clark WH Jr, Fraser MC, Stanger BZ, Green P, Donis-Keller H, Housman DE and Greene MH (1989) Mapping the gene for hereditary cutaneous malignant melanoma-dysplastic nevus to chromosome 1p. *N Engl J Med* **320**: 1367–1372

Bergman W, Watson P, de Jong J, Lynch HT and Fusaro RM (1990) Systemic cancer and the FAMMM syndrome. *Br J Cancer* **61**: 932–936

Bressac-de-Paillerets B, Soufir N, Chompret A, Bombled J, Demenais F, Spatz A and Avril M (1997) Germ-line mutations in p16 and CDK4 genes in 36 melanoma families. *Melanoma Res* **7**: S132

Cannon-Albright L, Goldgar D, Meyer L, Lewis C, Anderson D, Fountain J, Hegi M, Wiseman R, Petty E, Bale A, Olopade O, Diaz M, Kwiatkowski D, Piepkorn M, Zone J and Skolnick M (1992) Assignment of a locus for familial melanoma MLM, to chromosome 9p13–22. *Science* **258**: 1148–1152

Clark W, Reimer R, Greene M, Asnworth A and Masrangelo M (1978) Origin of familial malignant melanoma from hereditable melanocytic lesions: the BK mole syndrome. *Arch Dermatol* **114**: 732

Coleman K, Wautlet B, Morrissey D, Mulheron J, Sedman S, Brinkley P, Price S and Webster K (1997) Identification of CDK4 sequences involved in cyclin D1 and p16 binding. *J Biol Chem* **272**: 18869–18874

Dracopoli N and Fountain J (1996) CDKN2 mutations in melanoma. *Cancer Surv* **26**: 115–132

Fitzgerald M, Harkin D, Silva-Arrieta S, MacDonald D, Lucchina L, Unsal H, O'Neill E, Koh J, Finkelstein D, Isselbacher K, Sober A and Haber D (1996) Prevalence of germ-line mutations in p16, p19ARF and CDK4 in familial melanoma: analysis of a clinic based population. *Proc Natl Acad Sci USA* **93**: 8541–8545

Gemma A, Takenoshita S, Hagiwara K, Okamoto A, Spillare E, McMemamin M, Hussain S, Forrester K, Zariwala M, Xiong Y and Harris C (1996) Molecular analysis of the cyclin-dependent kinase inhibitor genes p15INK4B, p16INK4A, p18 and p19 in human cancer cell lines. *Intl J Cancer* **68**: 605–611

Goldstein AM and Tucker MA (1997) Screening for CDKN2A mutations in hereditary melanoma. *J Natl Cancer Inst* **89**: 676–678

Goldstein AM, Dracopoli NC, Ho EC, Fraser MC, Kearns KS, Bale SJ, McBride OW, Clark WH Jr and Tucker MA (1993) Further evidence for a locus for cutaneous malignant melanoma–dysplastic nevus (CMM/DN) on chromosome 1p, and evidence for genetic heterogeneity. *Am J Hum Genet* **52**: 537–550

Goldstein AM, Dracopoli NC, Engelstein M, Fraser MC, Clark WH Jr and Tucker MA (1994) Linkage of cutaneous malignant melanoma/dysplastic nevi to chromosome 9p, and evidence for genetic heterogeneity. *Am J Hum Genet* **54**: 489–496

Gruis NA, Bergman W and Frants RR (1990) Locus for susceptibility to melanoma on chromosome 1p. *N Engl J Med* **322**: 853–854

Guan K, Jenkins C, Li Y, Okeefe C, Noh S, Wu X, Zariwala M, Matera A and Xiong Y (1996) Isolation and characterisation of p19(INK4D), a p16-related inhibitor specific to CDK6 and CDK4. *Mol Biol Cell* **7**: 57–70

Harland MH, Meloni R, Gruis N, Pinney E, Brookes S, Spurr NK, Frischauf A-M, Bataille V, Peters G, Cuzick J, Selby P, Bishop DT and Newton Bishop J (1997) Germline mutations of the CDKN2 gene in UK melanoma families. *Hum Mol Genet* **6**: 2061–2067

Hirama T and Koeffler H (1995) Role of the cyclin-dependent inhibitors in the development of cancer. *Blood* **86**: 841–854

Hussussian CJ, Struewing JP, Goldstein AM, Higgins P, Ally DS, Sheahan MD, Clark WH Jr, Tucker MA and Dracopoli NC (1994) Germline p16 mutations in familial melanoma. *Nat Genet* **8**: 15–21

Kamb A, Shattuck-Eidens D, Eeles R, Liu Q, Gruis NA, Ding W, Hussey C, Tran T, Miki Y, Weaver-Feldhaus J, McClure M, Aitken JF, Anderson DE, Bergman W, Frants R, Goldgar DE, Green A, MacLennan R, Martin NG, Meyer LJ, Youl P, Zone JJ, Skolnick MH and Cannon-Albright LA (1994) Analysis of the p16 gene (CDKN2) as a candidate for the chromosome 9p melanoma susceptibility locus. *Nat Genet* **8**: 22–26

Liu L, Goldstein AM, Tucker MA, Brill H, Gruis NA, Hogg D and Lassam NJ (1997) Affected members of melanoma-prone families with linkage to 9p21 but lacking mutations in CDKN2A do not harbor mutations in the coding regions of either CDKN2B or $p19^{ARF}$. *Genes Chromosomes Cancer* **19**: 52–54

Lynch HT and Fusaro RM (1991) Pancreatic cancer and the familial atypical multiple mole melanoma (FAMMM) syndrome. *Pancreas* **6**: 127–131

MacGeoch C, Newton Bishop J, Bataille V, Bishop D, Frischauf A, Meloni R, Cuzick J, Pinney E and Spurr N (1994) Genetic heterogeneity in familial malignant melanoma. *Hum Mol Genet* **3**: 2195–2200

Mann G, Holland T, Grulet O-C, Rizos H and Kefford R (1997) Mutation and linkage analysis of CDKN2A and CDK4 in 119 Australian melanoma kindreds. *Am J Hum Genet* **61**: A73

Miller C, Yeon C, Aslo A, Mendoza S, Aytac U and Koeffler H (1997*a*) The p19INK4D cyclin dependent kinase inhibitor gene is altered in osteosarcoma. *Oncogene* **15**: 231–235

Nancarrow DJ, Palmer JM, Walters MK, Kerr BM, Hafner GJ, Garske L, McLeod GR and Hayward NK (1992) Exclusion of the familial melanoma locus (MLM) from the PND/D1S47 and MYCL1 regions of chromosome arm 1p in 7 Australian pedigrees. *Genomics* **12**: 18–25

Nancarrow D, Mann G, Holland E, Walker G, Beaton S, Walters M, Luxford C, Palmer J, Donald J, Weber J, Fountain J, Kefford R and Hayward N (1993) Confirmation of chromosome 9p linkage in familial melanoma. *Am J Hum Genet* **53**: 936–942

Nelson M, Thompson F, Emerson J, Aickin M, Adair L, Trent J, Leong S and Taetle R (1996) Clinical implications of cytogenetic abnormalities in melanoma. *Surg Clin N Am* **76**: 1257–1271

Newton J (1994) Genetics of melanoma. *Br Med Bull* **50**: 677–687

Parmiter A, Balaban G, Herlyn M, Clark W and Nowell P (1986) A t(1;19) chromosome translocation in three cases of human malignant melanoma. *Cancer Res* **46**: 1526–1529

Platz A, Hansson J, Mansson-Brahme E, Lagerlof B, Linder S and Lundqvist E (1997) Screening of germline mutations in the CDKN2A and CDKN2B genes in Swedish families with hereditary cutaneous melanoma. *J Nat Cancer Inst* **89**: 697–702

Ranada K, Hussussian C, Sikorski R, Varmus H, Goldstein A, Tucker M, Serrano M, Hannon G, Beach D and Dracopoli N (1995) Mutations associated with familial melanoma impair p16 INK4 function. *Nat Genet* **10**: 114–116

© Cancer Research Campaign 1999 

Sherr C (1996) Cancer cell cycles. *Science* **274**: 1672–1677

Sherr C and Roberts J (1995) Inhibitors of mammalian G1 cyclin-dependent kinases. *Genes Dev* **9**: 1149–1163

Shiohara M, Spirin K, Said JW, Gombart AF, Nakamaki T, Takeuchi S, Hatta Y, Morosetti R, Tasaka T, Seriu T, Bartram C, Miller CW, Tomonaga M and Koeffler HP (1996), Alterations of the cyclin-dependent kinase inhibitor p19$^{INK4D}$ is rare in hematopoietic malignancies. *Leukaemia* **10**: 1897–1900

Walker GJ, Nancarrow DJ, Walters MK, Palmer JM, Weber JL and Hayward NK (1994) Linkage analysis in familial melanoma kindreds to markers on chromosome 6p. *Intl J Cancer* **59**: 771–775

Walker G, Hussussian C, Flores J, Glendening J, Haluska F, Dracopoli N, Hayward N and Fountain J (1995) Mutations of the CDKN2/p16INK4 gene in Australian melanoma kindreds. *Hum Mol Genet* **4**: 1845–1852

Yang R, Serrano M, Slater J, Leung E and Koeffler HP (1996) Analysis of p16$^{INK4a}$ and its interaction with CDK4. *Biochem Biophys Res Commun* **218**: 254–259

Zindy F, Quelle D, Roussel M and Sherr C (1997) Expression of the p16INK4a tumor suppressor versus other INK4 family members during mouse development and ageing. *Oncogene* **15**: 203–211

Zuo L, Weger J, Yang Q, Goldstein A, Tucker M, Walker G, Hayward N and Dracopoli N (1996) Germline mutations in the p16INK4a binding domain of CDK4 in familial melanoma. *Nat Genet* **12**: 97–99

 *© Cancer Research Campaign 1999*
